# Role of Methoprene-Tolerant (Met) in Adult Morphogenesis and in Adult Ecdysis of *Blattella germanica*

**DOI:** 10.1371/journal.pone.0103614

**Published:** 2014-07-29

**Authors:** Jesus Lozano, Xavier Belles

**Affiliations:** Institut de Biologia Evolutiva, CSIC-Universitat Pompeu Fabra, Barcelona, Catalonia, Spain; U. Kentucky, United States of America

## Abstract

Juvenile Hormone (JH) represses metamorphosis of young instars in insects. One of the main players in hormonal signalling is Methoprene-tolerant (Met), which plays the role of JH receptor. Using the Polyneopteran insect *Blattella germanica* as the model and RNAi for transcript depletion, we have confirmed that Met transduces the antimetamorphic signal of JH in young nymphs and plays a role in the last nymphal instar moult in this species. Previously, the function of Met as the JH receptor had been demonstrated in the Eumetabola clade, with experiments in Holometabola (in the beetle *Tribolium castaneum*) and in their sister group Paraneoptera (in the bug *Pyrrhocoris apterus*). Our result shows that the function of Met as JH receptor is also conserved in the more basal Polyneoptera. The function of Met as JH transducer might thus predate the evolutionary innovation of metamorphosis. Moreover, expression of Met was also found in last nymphal instar of *B. germanica*, when JH is absent. Depletion of Met in this stage provoked deficiencies in wing growth and ecdysis problems in the imaginal moult. Down-regulation of the ecdysone-inducible gene E75A and Insulin-Like-Peptide 1 in these Met-depleted specimens suggest that Met is involved in the ecdysone and insulin signalling pathways in last nymphal instar, when JH is virtually absent.

## Introduction

In essence, insect metamorphosis is regulated by the interplay of two hormones, 20-hydroxyecdysone (20E), which triggers the successive moults throughout the life cycle, and juvenile hormone (JH), which represses the transition to the adult stage [1]. From an evolutionary point of view, it is worth noting that this essential endocrine regulation is conserved in the species closest to the ancestral state, where juvenile stages are similar to the adult (hemimetabolan insects), as well as in more derived species, where juvenile stages can be extremely divergent with respect to the adult stage (holometabolan insects) [1].

While molecular mechanisms regulating the action of 20E have largely been unveiled, those underlying the action of JH have remained almost a complete mystery until recent years, when the transcription factor Methoprene-tolerant (Met) has been proclaimed as the JH receptor [2]. In 1986, Met was discovered to be a factor that determines resistance to the toxic effects of Methoprene, a JH analogue, in some *Drosophila melanogaster* strains [3]. Later, Met was identified as a member of the basic helix-loop-helix Per-ARNT-Sim (bHLH-PAS) family of transcription factors [4], which are critical regulators of gene expression networks underlying many essential physiological and developmental processes [5]. Subsequently, Miura et al. [6] showed that *D. melanogaster* Met protein could bind JH with a very high affinity (Kd of 5.3 nM), providing the first clear indication that Met might play the role of JH receptor. More recently, it has been observed that depletion of Met mRNA levels with RNAi in early larval stages of the holometabolan species *T. castaneum* triggers precocious pupal morphogenesis [7], [8], which showed that Met is involved in antimetamorphic JH signal transduction. RNAi studies also demonstrated the JH-transducing role of Met in the hemimetabolan species *Pyrrhocoris apterus* [9]. Using Met of *T. castaneum*, Charles et al. [10] confirmed that the JH binding affinity is high (Kd of 2.9 nM in this case), that the PAS-B motif of Met is both necessary and sufficient to bind JH, and that when JH binds to a Met moiety in a Met-Met homodimer, the homodimer dissociates and JH+Met binds to another bHLH-PAS protein called Taiman.

In the meantime, it was found that the JH signal downstream of Met is transduced by Krüppel-homolog 1 (Kr-h1), a transcription factor with a DNA binding domain consisting of eight zinc fingers. The function of Kr-h1 as a transducer of the antimetamorphic action of JH was demonstrated through RNAi experiments in holometabolan species, such as *D. melanogaster* [11] and *T. castaneum* [12], as well as in hemimetabolan species, such as the cockroach *Blattella germanica* [13] and the bugs *P. apterus* and *Rhodnius prolixus* [9]. In all cases, depletion of Kr-h1 mRNA levels in pre-final juvenile stages triggered precocious metamorphosis.

From a mechanistic point of view, results obtained in NIAS-Bm-aff3 cells from the silkworm *Bombyx mori* [14] are specially relevant because they revealed the presence of a JH response element, which was named *k*JHRE, in the promoter region of *B. mori* Kr-h1. Interestingly, *k*JHRE contains an E-box to which bHLH-PAS proteins may bind, and reporter assays in mammalian HEK293 cells (which presumably lack JH signalling genes) showed that while *k*JHRE-specific reporter activity was triggered in the presence of JH when the complete open reading (ORF) frame of *B. mori* Met was expressed, this activation was higher when Taiman ORF was coexpressed with Met. Therefore, these results suggest that Met and Tai jointly interact with the *k*JHRE located in the Kr-h1 promoter region and that this interaction is JH-dependent [14]. Equivalent experiments carried out on the Tc81 embryonic cell line of *T. castaneum* [15] led to the identification of the same *k*JHRE, this time located in both the promoter region and in the first intron of *T. castaneum* Kr-h1. Moreover, reporter assays performed with HEK293 cells also showed that *k*JHRE reporter activity was triggered when Met was expressed in the presence of JH and the activation was enhanced when Taiman was coexpressed with Met [15]. Collectively, the data presents clear evidence that Met plays the role of JH receptor and, therefore, in metamorphosis repression, at least in Paraneopteran insects such as the bug *P. apterus* [9] and in Holometabola such as the beetle *T. castaneum* [7], [8].

The present work aims to extend the investigation into the JH receptor role of Met in metamorphosis to Polyneopteran insects, taking the cockroach *B. germanica* as model. Moreover, our preliminary experiments showed that Met is also expressed in last nymphal instar of this cockroach, intriguingly when JH is absent. A second purpose of the present work was, then, to study the function of Met in this stage.

## Materials and Methods

### Insects

The *B. germanica* specimens used in the experiments were from a colony reared in the dark at 30±1°C and 60–70% relative humidity. Freshly ecdysed female nymphs were selected and used at the chosen ages. Prior to injection treatments, dissections and tissue sampling, the specimens were anaesthetized with carbon dioxide.

### RNA Extraction and retrotranscription to cDNA

We carried out total RNA extraction from the whole body (excluding the digestive tube to avoid intestine parasites) or specific tissues using the miRNeasy extraction kit (QIAGEN). A sample of 500-ng from each RNA extraction was treated with DNase (Promega) and reverse transcribed with first Strand cDNA Synthesis Kit (Roche) and random hexamers primers (Roche). RNA quantity and quality was estimated by spectrophotometric absorption at 260 nm using a Nanodrop Spectrophotometer ND-1000 (NanoDrop Technologies).

### Cloning and sequencing of BgMet

BgMet mRNA was obtained from a *B. germanica* transcriptome obtained with RNA extracts from whole body of penultimate instar female nymphs and sequenced with a 454 Junior sequencer (Roche, Barcelona, Spain) at the Technical and Scientific Services of the Biomedical Research Park of Barcelona (PRBB). Transcriptome sequences were validated with RT-PCR using specific primers and cDNA from penultimate instar female nymphs of *B. germanica* as a template. Further 3′ and 5′ rapid amplification of cDNA ends (RACE, Ambion) allowed us to obtain a practically complete full length ORF of BgMet. All PCR products were subcloned into the pSTBlue-1 vector (Novagen) and sequenced.

### Determination of mRNA levels by quantitative real-time PCR

Quantitative real time PCR (qRT-PCR) reactions were carried out in triplicate in an iQ5 Real-Time PCR Detection System (Bio-Rad Laboratories), using SYBR Green (Power SYBR Green PCR Master Mix; Applied Biosystems). A template-free control was included in all batches. The primers used to detect mRNA levels are detailed in Table S1. The efficiency of each set of primers was first validated by constructing a standard curve through four serial dilutions. Levels of mRNA were calculated relative to BgActin-5c (Accession number AJ862721) expression, using the Bio-Rad iQ5 Standard Edition Optical System Software (version 2.0). Results are given as copies of mRNA per 1000 copies of BgActin-5c mRNA or as standardized relative expression, setting controls levels to 1.0.

### Treatments in vivo with juvenile hormone III

Freshly emerged last instar female nymphs of *B. germanica* were topically applied in dorsal abdomen with JH III (Sigma-Aldrich), which is the native JH of B. *germanica* [16], at a dose of 20 µg diluted in 1 µL of acetone. Controls received 1 µL of acetone alone. The commercial JH III is a mixture of isomers containing *ca*. 50% of the biologically active (10*R*)-JH III, thus the active dose applied was around 10 µg per specimen.

### RNA interference

The detailed procedures for RNAi experiments were as described previously [17]. The two dsRNAs used for BgMet targeting as well as the primers to generate the corresponding templates are summarized in Table S1. The fragments were amplified by PCR and cloned into the pSTBlueTM-1 vector. A 307-bp sequence from *Autographa californica* nucleopoyhedrovirus was used as control dsRNA (dsMock). The dsRNAs were prepared as reported elsewhere [17]. A volume of 1 µL of dsRNA solution (3 µg/µL, unless stated otherwise) was injected into the abdomen of specimens at chosen ages and stages with a 5-µL Hamilton microsyringe. Control specimens were treated with the same dose and volume of dsMock.

## Results

### *B. germanica* has a conserved orthologue of Met

**Figure 1:**
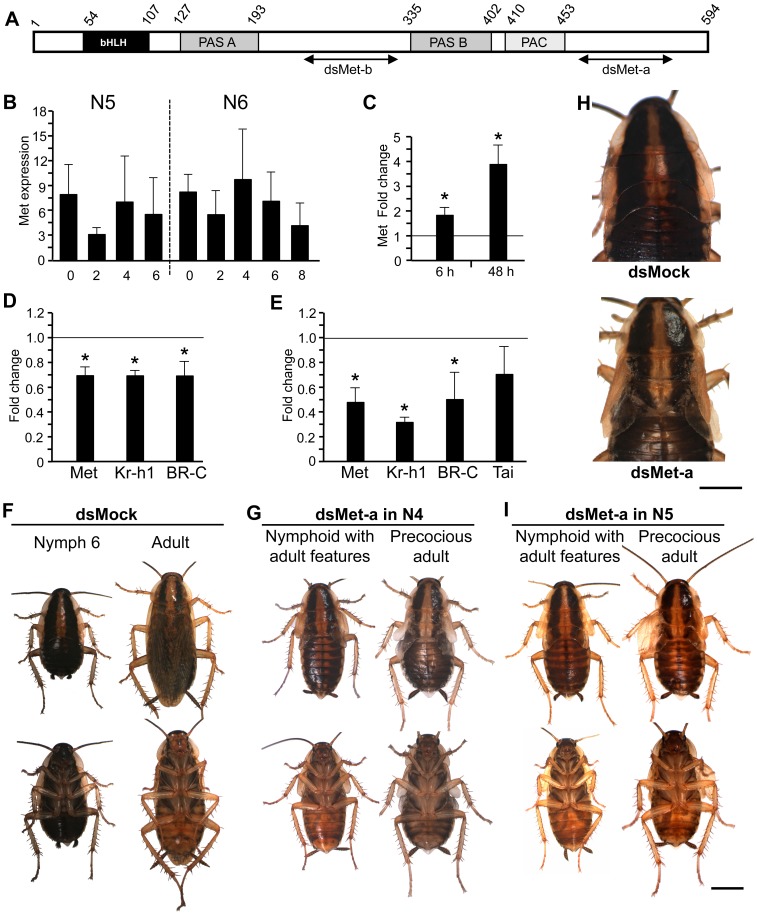
Structure, expression and function of BgMet in *Blattella germanica* metamorphosis. (A) Organization of BgMet protein in different domains; also indicated are the regions were the two dsMet used in RNAi studies were designed. (B) Expression of BgMet mRNA in whole body of female nymphs in penultimate (N5) and last (N6) instar. (C) Effect of JH III treatment (20 µg) on BgMet expression; JH was topically applied in freshly emerged N6, and BgMet mRNA levels were measured 6 and 48 h later. (D) Effects, at transcript level, of dsMet-a treatment in N4; N4 females received two 3-µg doses of dsMet-a, one on N4D0 and the other on N4D3, and transcript levels (of Met, Kr-h1 and BR-C) were measured on N4D5; controls received an equivalent treatment with dsMock. (E) Effects, at transcript level, of dsMet-a treatment in N5; the experimental design was equivalent to that used in N4, with a double treatment, one on N5D0 and the other on N5D3; transcript levels (of Met, Kr-h1, BR-C and Tai) were measured on N5D6. (F–I) Dorsal and ventral view of specimens resulting from dsMet-a treatment in N4 and N5; normal last nymphal instar and adult obtained from dsMock treatments, either in N4 or N5 (F); nymphoid (instead of N6) with adult features, and precocious adult obtained from dsMet-a treatments in N4 (G), detail of the enlarged lateral expansions in T2 and T3 of a nymphoid with adult features compared with a control N6 (H), nymphoid with adult features, and precocious adult obtained from dsMet-a treatments in N5 (I). Each point of quantitative data in histograms represents 4 biological replicates and results are expressed as the mean ± SEM; data in A represent copies of BgMet mRNA per 1000 copies of BgActin-5c mRNA; data in C, D and E are normalized against the dsMock-treated samples (reference value = 1), and the asterisk indicates statistically significant differences with respect to controls (p<0.05), according to the REST software tool [29]. Photomicrographs in F–I were taken with a Zeiss DiscoveryV8 Stereo microscope with an AxioCam MRc digital camera; scale bars in F, G, I = 3 mm and in H = 2 mm.

Combining BLAST search in *B. germanica* transcriptomes available in our laboratory and PCR strategies, we obtained a cDNA of 1783 bp (GenBank accession number HG965209) whose conceptual translation rendered a 594 amino acid protein with sequence similarity to Met proteins that we called BgMet. The BgMet sequence contains the bHLH, PAS A, PAS B and PAC (PAS-associated C-terminal region) motifs that are typical of Met proteins (Figure 1A) and top BLAST scores were obtained from Met orthologues of other insects. The highest level of overall amino acid sequence identity (47%) was found with the Met orthologue of the firebrat *Thermobia domestica*, whereas the lowest level (29%) was with Met1 of *B. mori* (Figure S1).

### Expression of Met is maintained during the last two nymphal instars of *B. germanica*

BgMet transcript levels fluctuate between 3 and 10 mRNA copies per 1000 copies of actin mRNA, either in the penultimate (N5) or last (N6) nymphal instars (Figure 1B). Expression in N5 is not surprising because JH, which represses metamorphosis in young nymphs, is produced at significant levels throughout this stage [16]. However, BgMet is also expressed in N6 when there is no JH [16] to be transduced. To test whether BgMet is JH-inducible, we treated freshly emerged sixth instar female nymphs with JH III, the naturally occurring JH homologue in *B. germanica* [16], and measured BgMet transcript levels 6 and 48 h after treatment. Results showed that JH up-regulates BgMet expression and that the stimulatory effect increases with time (Figure 1C). JH-treated specimens not used for transcript measurements moulted into supernumerary nymphs, as observed in previous studies [18].

### Depletion of Met in young nymphal instars triggers precocious metamorphosis

Involvement of Met in *B. germanica* metamorphosis was studied by RNAi approaches using a double-stranded RNA (dsRNA) which targets BgMet (dsMet-a) (Figure 1A). In a first set of experiments, we injected two 3-µg doses of dsMet-a in antepenultimate (N4) nymphal instar females, one when freshly emerged (N4D0) and the other on day 3 (N4D3). Controls received an equivalent treatment with dsMock. Transcript measurements carried out on N4D5 indicated that BgMet mRNA levels were already significantly reduced (*ca.* 30%) in dsMet-a-treated specimens (Figure 1D). At the same time, we measured the expression of Kr-h1 and BR-C, two JH-dependent genes, and both were also down-regulated at similar levels than Met (Figure 1D). In a second set of experiments we treated N5 female instar following an experimental design equivalent to that used for N4 nymphs, with a double 3-µg treatment, one on N5D0 and the other on N5D3. Transcript levels of Met were measured on N5D6, and resulted to be significantly reduced (*ca.* 50%) (Figure 1E). We also measured the expression of Kr-h1, BR-C and that of Tai, as a putative partner of Met for JH reception and signal transduction. Kr-h1 and BR-C expression was significantly down-regulated (*ca.* 70 and 50%, respectively), whereas Tai mRNA levels did tend to be lower, the differences with respect to dsMock-treated specimens were not statistically significant (Figure 1E).

At the phenotypic level, we first analysed the specimens that received a double treatment of dsMet-a or dsMock in N4. All dsMock-treated specimens (n = 27) moulted to N5, then to N6 and to adult, all stages showing normal morphological features (Figure 1F). As regards to the dsMet-a-treated specimens (n = 54), 9 of them (17%) died during N4 or N5, often during moulting, just before or while ecdysing. The remaining 45 specimens that survived N4 and N5 stages produced the following different phenotypes after the next moult: 8 specimens (18%) died during the next ecdysis, presenting fundamentally nymphal features, but with flexible and somewhat enlarged lateral expansions in the mesonotum (T2) and metanotum (T3) (Figure S2A); 7 specimens (16%) showed a phenotype that we categorised as nymphoid with adult features, having a general shape similar to an N6 nymph but exhibiting adult yellowish colouration and enlarged and membranous wing-like lateral expansions in T2 and T3 (Figure 1 G and H); 11 specimens (24%) that we categorised as precocious adults, exhibiting a general shape and yellow colouration similar to an adult but with tegmina and wings that were membranous and flexible yet underdeveloped (Figure 1G); and 19 specimens (42%) showing the morphology of a normal N6. In turn, these 19 specimens underwent the imaginal moult; 10 of them died between apolysis and ecdysis, 4 moulted to an adult with the tegmina and wings correctly patterned but not well extended, and also with deformed hind tibiae (Figure S2B), and the remaining 5 moulted to normal adults (results summarised in Table S2).

Then, we analysed the specimens that received a double treatment of dsMet-a or dsMock in N5. All dsMock-treated specimens (n = 19) moulted to normal N6 and then to normal adults. None of the dsMet-a-treated specimens (n = 35) died during N5 or N6. After the moult from N5, 5 out of the 35 specimens (14%) were nymphoids with adult features (Figure 1I), 1 specimen (3%) had a precocious adult morphology (Figure 1I) and 29 specimens (83%) were normal N6 nymphs. Of the 29 N6 females that underwent the imaginal moult, 5 of them died between apolysis and ecdysis, 12 moulted to adults with the tegmina and wings correctly patterned but not well extended (as in Figure S2B), and the remaining 12 moulted to normal adults (Table S2).

To assess the specificity of dsMet-a, we tested an alternative Met-targeting dsRNA (dsMet-b) designed in another region of the BgMet sequence (Figure 1A). We carried out experiments equivalent to those performed with dsMet-a in N4 and N5 nymphs, and the results were equivalent in phenotypical terms (Figure S3A and Table S2). We mistakenly included a male nymph in the experiments in N4, which moulted to N5, and then to a practically perfect precocious adult, including correctly patterned tergal glands, similar to those obtained after Kr-h1 depletion [13] (Figure S3B). Depletion of Met expression and the effects on other transcripts (Kr-h1, BR-C and Taiman) in N5 treatments were similar to those obtained with dsMet-a (Figure S3C).

### Depletion of Met in the last nymphal instar delays the adult moult and provokes ecdysis deficiencies

**Figure 2:**
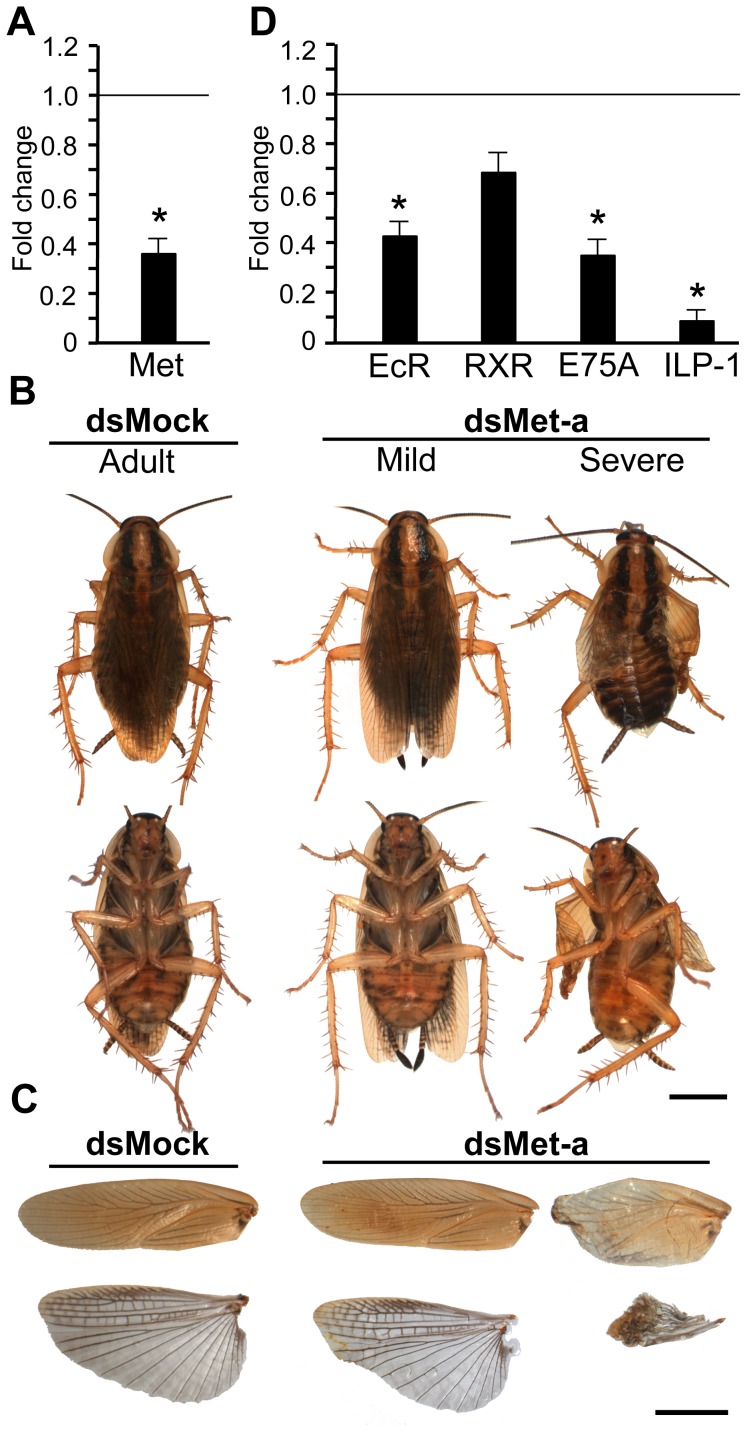
Effects of BgMet depletion in last nymphal stage of *Blattella germanica*. (A) Effects at transcript level; N6 females received two 3-µg doses of dsMet-a, one on N6D0 and the other on N6D3, and Met mRNA levels were measured on N6D6; controls received an equivalent treatment with dsMock. (B) Dorsal and ventral view of specimens resulting from dsMet-a treatment in N6; normal adult obtained from dsMock treatments; mild and severe adult phenotypes obtained after Adult N6 with adultoid features, mild and severe, obtained from dsMet-a treatments. (C) Tegmina and membranous wings corresponding to normal adult obtained after dsMock treatments, and to adults treated with dsMet-a showing the mild and the severe phenotype. (D) Effects of Met depletion on N6 on the expression of EcR, RXR, E75A and ILP-1 measured on N6D6. In A and D, each point represents 4 biological replicates and results are expressed as the mean ± SEM; data are normalized against the dsMock-treated samples (reference value = 1), and the asterisk indicates statistically significant differences with respect to controls (p<0.05), according to the REST software tool [29]. Photomicrographs in B–C were with a Zeiss DiscoveryV8 Stereo microscope with an AxioCam MRc digital camera; scale bars = 3 mm.

The expression of Met in N6 (Figure 1B), when JH is absent, lead us to consider Met functions in this stage. To address this question, we administered two doses of dsMet-a to N6 females, one on N6D0 and the other on N6D3, whereas controls received an equivalent treatment with dsMock. Transcript measurements performed on N6D6 revealed that BgMet mRNA levels were significantly reduced (*ca.* 65%) in dsMet-a-treated specimens (Figure 2A). At the phenotypic level, all specimens treated with dsMock (n = 14) moulted to normal adults (Figure 2B), with the tegmina and membranous wings well extended (Figure 2C), and they spent 8 days in N6, as usual in our rearing conditions. The dsMet-a-treated specimens (n = 28) spent an average of 9 days in N6 and a number of deficiencies arose at the imaginal moult. Five specimens (18%) died between apolysis and ecdysis. Ten (36%) moulted to adults with abnormalities in the tegmina and membranous wings, ranging from relatively mild (both slightly smaller and the tegmina somewhat curled at the tip) to severe (both tegmina and membranous wings severely wrinkled and reduced) (Figure 2B). Finally, 13 specimens (46%) moulted to normal adults (Table S2).

Given that the phenotypes obtained pointed to moulting problems, we wondered whether the expression of genes of the ecdysone signalling pathway could be affected. We measured mRNA levels of the components of the ecdysone receptor in dsMet-a-treated specimens; results showed that those of EcR were significantly lower (*ca.* 60%) than controls, whereas those of RXR tended to be lower but differences with respect to controls were not statistically significant. The ecdysone inducible gene E75A was significantly down-regulated (*ca.* 65%) (Figure 2D). As moulting and wing size problems also suggested that insulin pathway might be affected, we measured the *B. germanica* orthologue of Insulin-Like-Peptide 1 (ILP-1), which is the most highly expressed ILP in *B. germanica* brain (J. L. Maestro, unpublished data). ILP-1 mRNA levels in dsMet-a-treated specimens were significantly, and dramatically, lower (*ca.* 90%) than in control specimens (Figure 2D).

## Discussion

*B. germanica* Met is the first Met sequence reported in Dictyoptera, a Polyneopteran within the Pterygota subclass of hemimetabolan insects. In terms of overall percentage of identity, BgMet is closer to *T. domestica* (an Apterygota ametabolan species) than to the phylogenetically closer Pterygota hemimetabolan *L. migratoria*. As expected, the homologous sequences of Diptera and Lepidoptera, the most modified orders of Pterygota Holometabola, are the most divergent with respect to BgMet.

Expression of BgMet in the penultimate (N5) nymphal instar, when JH is present, is not surprising. In this instar, we propose that Met transduces the JH signal that represses adult morphogenesis, as Met depletion during this stage triggers precocious metamorphosis at the next moult. When dsMet was administered in the antepenultimate (N4) nymphal instar the adult characters appeared after two moults. Similar findings relating to the precocious apparition of adult characters after depleting Met in pre-final juvenile stages have been reported in both hemimetabolan (*P. apterus*) and holometabolan (*T. castaneum*) species [7]–[9]. These data suggest that insects must attain a minimum critical size to activate adult morphogenesis, and that in our study the *B. germanica* N4 did not attain such a size and therefore moulted to N5 despite dsMet treatment. Moreover, adult features triggered by Met depletion were more apparent and with higher penetrance when the specimens were treated in earlier stages, N4, compared to N5, possibly because the experimental design allowed more time for tissue growth and development and for more effective Met depletion. These observations are in line with the recently proposed concept that insects acquire competence to metamorphose only in late nymphal or larval instars [19].

Met depletion in *B. germanica* provoked significant mortality and the formation of precocious, although imperfect, adults in what should be N6. This contrasts with results obtained after depleting Kr-h1 in young instars which did not provoke mortality and induced the precocious formation of practically perfect adults [13]. The precocious imperfect adult morphogenesis and higher mortality obtained after depleting Met may be due to the fact that Met is upstream of Kr-h1 and its depletion could also affect other pathways that do not directly involve Kr-h1. Secondly it may also be because the level of Met depletion, and the concomitant decrease in Kr-h1 mRNA levels, was less than that achieved when directly depleting Kr-h1 [13].

Intriguingly, BgMet was also expressed in the last (N6) nymphal instar of *B. germanica*, when JH is absent [16], and this led us to study its possible functions in this instar. When Met mRNA levels were depleted in N6, the length of the instar was extended by one day with respect to the controls and ecdysis to the adult stage was deficient in approximately half the cases. This occurred especially because the insects were incapable of fully shedding the exuvium, which resulted in deficiencies in wing extension and other, mainly mechanical problems. Previous studies have shown that Met depletion in late larval stages of *T. castaneum* and *B. mori* provokes a certain level of mortality and ecdysis complications [7], [8], [20], [21]; in *B. mori*, moreover, Met-depleted specimens moulted on average one day later than controls [20]. In our experiments, expression of genes involved in the ecdysone signalling pathway, such as EcR, RXR and E75A, was reduced in Met-depleted specimens, suggesting that Met is involved in this pathway. Indeed, the phenotypes obtained are reminiscent of the less affected phenotypes obtained after depleting EcR-A in the last nymphal instar of *B. germanica* [22]. Bitra and Palli [23] provided other cues that point to this involvement when they demonstrated that Met physically interacts with EcR-USP in *D. melanogaster*, while Guo et al. [20] reported that Met is required for maximum activity of the ecdysone signalling pathway during metamorphosis of *B. mori*. The malformed adult phenotypes observed after moulting problems, especially in terms of wrinkled wings and deformed tibiae and which were obtained when depleting Met in N4 and N5, suggest the hypothesis that Met is also involved in the ecdysone signalling pathway in *B. germanica*, a hypothesis that would require further study to elucidate the mechanistic aspects.

The delayed onset of moulting and ecdysis plus wing size complications, might also be caused by metabolic and growth deficiencies related to the Insulin/IGF-1 signalling (IIS) pathway. Our experiments showed that expression of *B. germanica* ILP-1 was dramatically reduced in Met-depleted specimens, which suggests that Met enhances ILP-1 transcription without the contribution of JH. JH and Met involvement in the IIS pathway has already been reported in *T. castaneum* adults [24]. Depletion of Met in adult females reduced the expression of two of the four *T. castaneum* ILPs (ILP-2 and -3, although *T. castaneum* ILPs cannot be homologated to *B. germanica* ILP-1) in brain and fat body. In the fat body, vitellogenin transcription, which is JH-dependent, was also reduced in Met-depleted specimens and the authors showed that JH-induced vitellogenin expression in the fat body is mediated by ILP signalling [24]. More recently, the same research group has shown that depletion of JH-acid-methyltransferase (a key enzyme for JH synthesis), Met or ILP-2 mRNA in starved adult males of *T. castaneum* decreased lipid and carbohydrate metabolism and extended their survival [25]. In any case, the possible involvement of Met in the insulin pathway and in the last instar nymph of *B. germanica*, when JH is absent, would merit further study to elucidate the underlying mechanisms.

As a main conclusion, our results show that Met transduces the JH signal and, therefore, represses metamorphosis in the cockroach *B. germanica*, a representative of Polyneoptera. This function of Met had previously been demonstrated in Holometabola (*T. castaneum*) and Paraneoptera (*P. apterus*), thus meaning that it is present across Eumetabola, according the phylogeny of the extant hexapod orders reported by Wheeler et al. [26]. Our results extend this function to the Polyneoptera, thereby encompassing all Neoptera. Met is also found in the firebrat *T. domestica* [9] but its function in this basal Zygentoma species is yet unknown. Given that *T. domestica* is ametabolan, Met function in this species cannot be related to metamorphosis, but it could, however, be related to JH transduction for vitellogenin production and oocyte growth, as occurs in the hemipteran *P. apterus*, according to the study of Smykal et al. [27]. If so, then the function of Met as JH signal transducer would predate the emergence of winged insects and the evolutionary innovation of metamorphosis [28].

## Supporting Information

Figure S1 **Comparison of BgMet with other insect Met protein sequences.** In addition to the percentage of overall identity, we indicate the percentage of identity for each of the characteristic domains of the protein. The species included (and the respective code and accession number in GenBank) are: *Thermobia domestica* (TdMet, AEW22978), *Rhodnius prolixus* (RpMet, AEW22977), *Locusta migratoria* (LmMet, AHA42531), *Tribolium castaneum* (TcMet, BAG71980), *Acyrtosiphon pisum* (ApMet, XP_003246905), *Aedes aegypti* (AaMet, AAW82472), *Pyrrhocoris apterus* (PaMet, AEW22976), *Drosophila melanogaster* Germ cell-expressed (DmGCE, NP_511160), *Bombyx mori* (BmMet 2, BAJ05086), *Drosophila melanogaster* Methoprene-tolerant (DmMet, NP_511126), *Bombyx mori* (BmMet 1, NP_001108458). * indicate that the sequence lacks the region between the initial Met and the bHLH domain and a part of the latter, and ** that the sequence lacks the region between the initial Met and the bHLH domain, but the latter is complete.
(TIF)

Figure S2 **Mild phenotypes obtained after treating** ***Blattella germanica*** **N4 with dsMet-a.** (A) Dorsal and ventral view of a specimen that died during the ecdysis to N6; the detail shows that the lateral expansions in the mesonotum and metanotum are somewhat enlarged and apparently flexible (indicated with an arrow in the detail). (B) Dorsal and ventral view of a specimen that moulted from N6 to adult with the tegmina and wings correctly patterned but not well extended, and with the hind tibiae deformed.
(TIF)

Figure S3 **Effects of Met depletion with dsMet-b in** ***Blattella germanica*** **.** (A) Dorsal and ventral view of a precocious adult obtained after treating with dsMet-b in N5. (B) Dorsal and ventral view of a male precocious adult obtained after treating with dsMet-b in N4. (C) Effects, at transcript level, of dsMet-b treatment in N5; insects received a 3-µg dose in N5D0 and another on N5D3; transcript levels (of Met, Kr-h1, BR-C and Tai) were measured on N5D6. Each point represents 4 biological replicates and results are expressed as the mean ± SEM; data are normalized against the dsMock-treated samples (reference value = 1), and the asterisk indicates statistically significant differences with respect to controls (p<0.05), according to the REST software tool [29].
(TIF)

Table S1 **Primers used to detect transcript levels by qPCR and those used to prepare the dsRNAs for RNAi experiments.**
(PDF)

Table S2 **Summary of the effects of Met depletion at phenotypic level in the experiments treating with dsMet-a or dsMet-b and at the stages N4, N5 or N6.** In all cases the corresponding dsRNA was administered in two 3 µg-doses, one on day 0 and the other on day 3 of the given instar. Controls were equivalently treated with dsMock. Methodological details are described in the main text.
(PDF)
