# Supplementary figures and images for: Role of Methoprene-Tolerant (Met) in Adult Morphogenesis and in Adult Ecdysis of Blattella germanica
Source: PLoS One. 2014 Jul 29;9(7):e103614. doi: 10.1371/journal.pone.0103614 (PMC4114754; doi:10.1371/journal.pone.0103614)

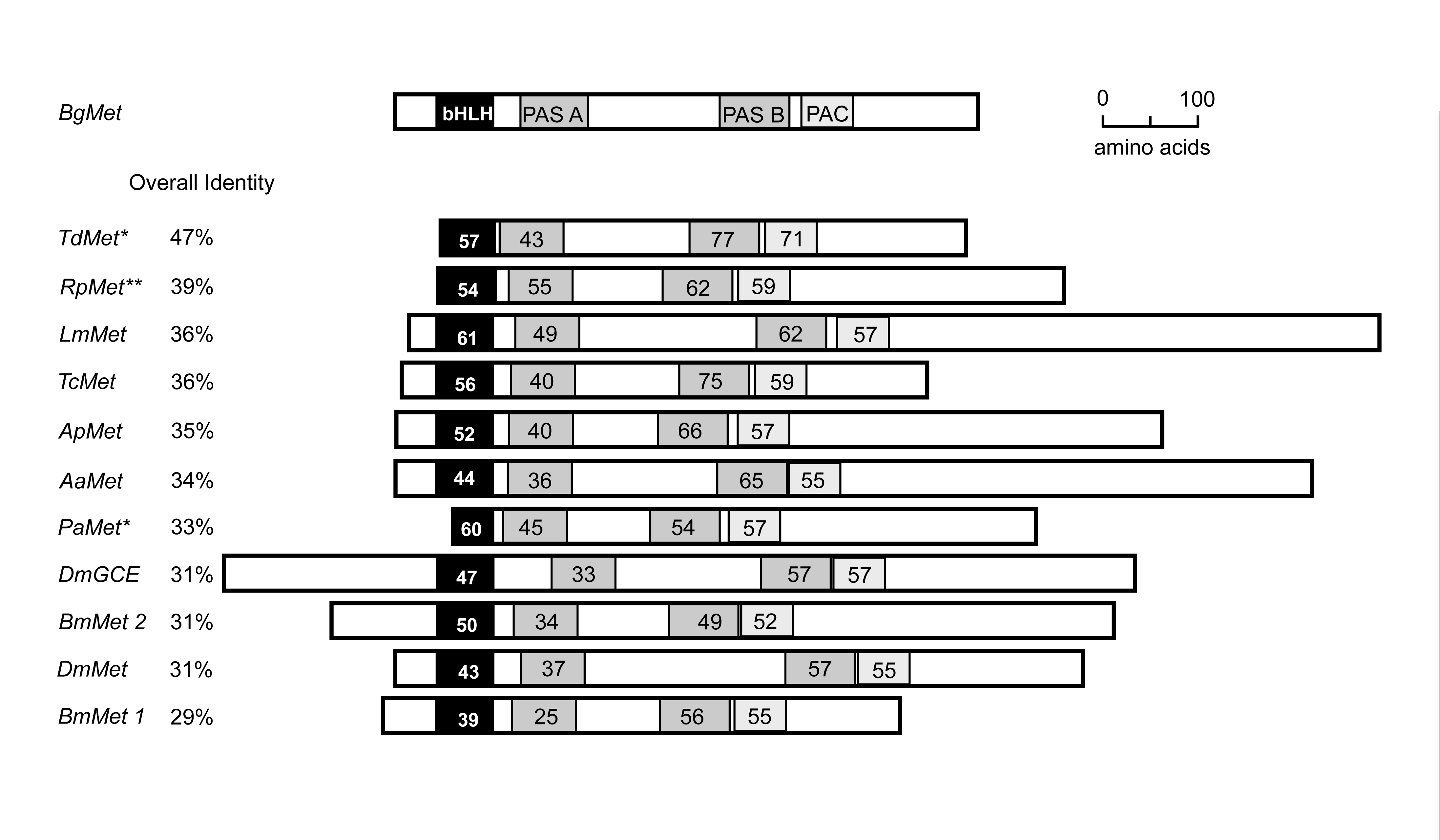

Supplement: Figure S1 — Comparison of BgMet with other insect Met protein sequences. In addition to the percentage of overall identity, we indicate the percentage of identity for each of the characteristic domains of the protein. The species included (and the respective code and accession number in GenBank) are: Thermobia domestica (TdMet, AEW22978), Rhodnius prolixus (RpMet, AEW22977), Locusta migratoria (LmMet, AHA42531), Tribolium castaneum (TcMet, BAG71980), Acyrtosiphon pisum (ApMet, XP_003246905), Aedes aegypti (AaMet, AAW82472), Pyrrhocoris apterus (PaMet, AEW22976), Drosophila melanogaster Germ cell-expressed (DmGCE, NP_511160), Bombyx mori (BmMet 2, BAJ05086), Drosophila melanogaster Methoprene-tolerant (DmMet, NP_511126), Bombyx mori (BmMet 1, NP_001108458). * indicate that the sequence lacks the region between the initial Met and the bHLH domain and a part of the latter, and ** that the sequence lacks the region between the initial Met and the bHLH domain, but the latter is complete. (TIF) [file pone.0103614.s001.tif]

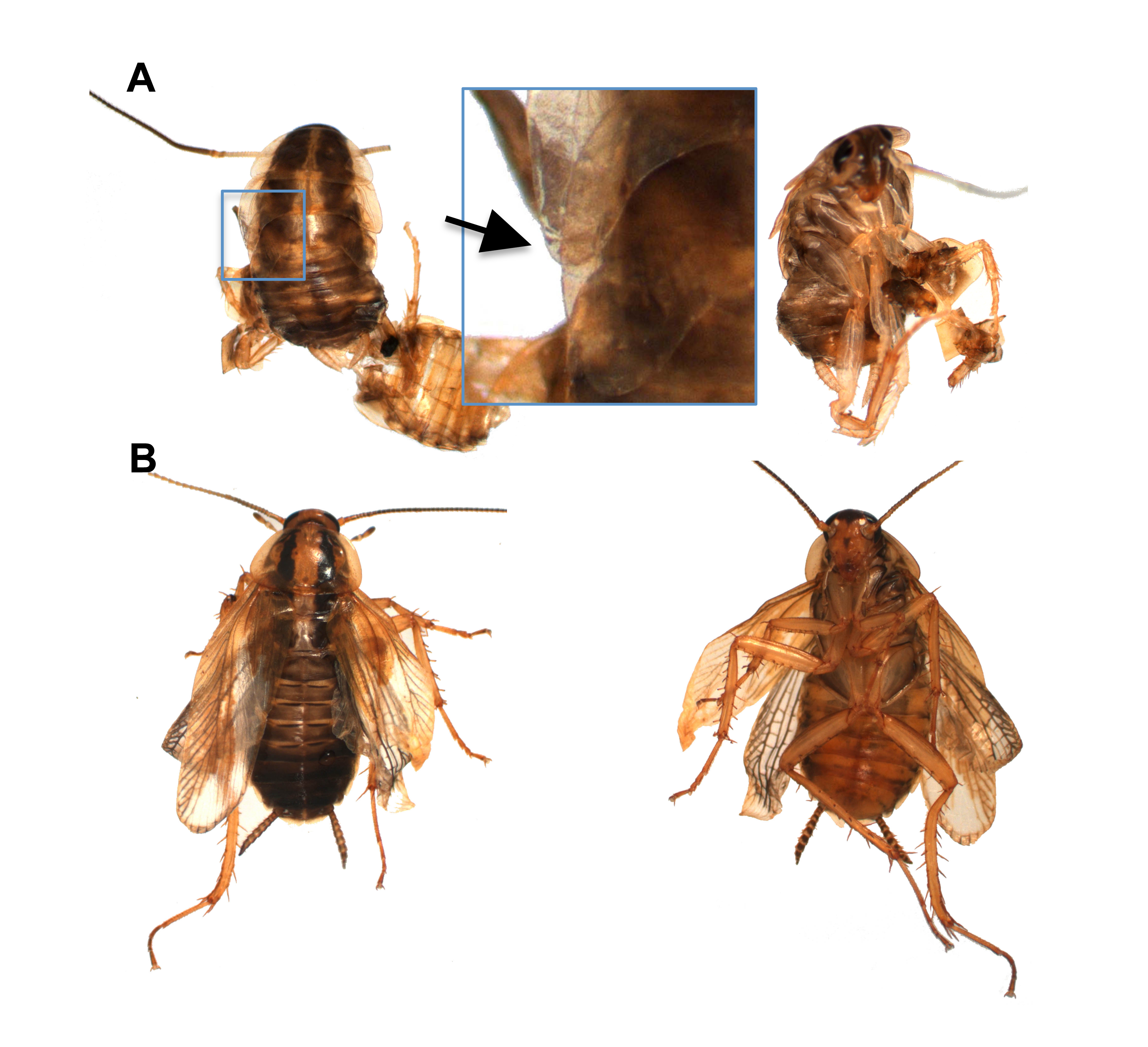

Supplement: Figure S2 — Mild phenotypes obtained after treating Blattella germanica N4 with dsMet-a. (A) Dorsal and ventral view of a specimen that died during the ecdysis to N6; the detail shows that the lateral expansions in the mesonotum and metanotum are somewhat enlarged and apparently flexible (indicated with an arrow in the detail). (B) Dorsal and ventral view of a specimen that moulted from N6 to adult with the tegmina and wings correctly patterned but not well extended, and with the hind tibiae deformed. (TIF) [file pone.0103614.s002.tif]

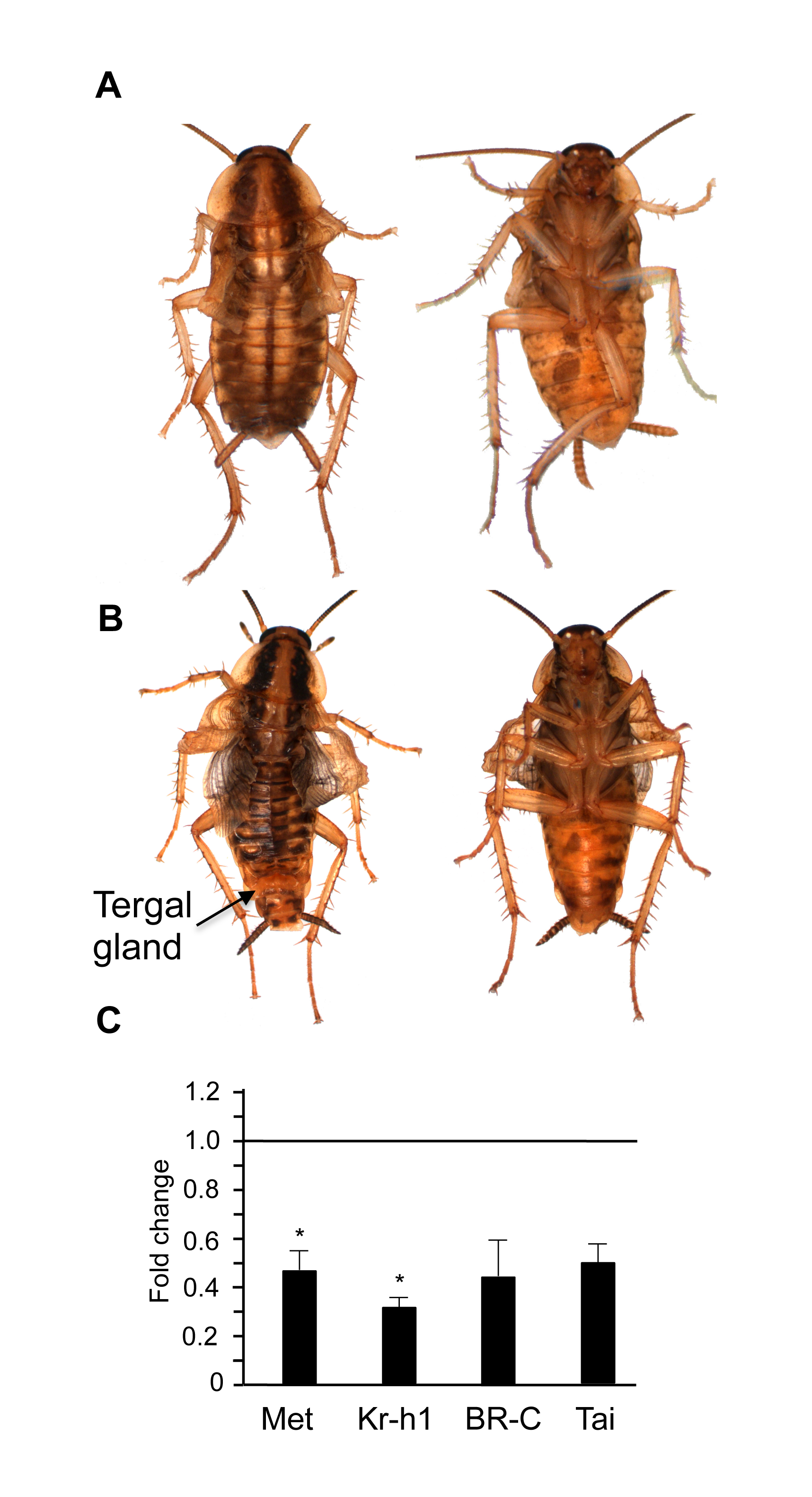

Supplement: Figure S3 — Effects of Met depletion with dsMet-b in Blattella germanica . (A) Dorsal and ventral view of a precocious adult obtained after treating with dsMet-b in N5. (B) Dorsal and ventral view of a male precocious adult obtained after treating with dsMet-b in N4. (C) Effects, at transcript level, of dsMet-b treatment in N5; insects received a 3-µg dose in N5D0 and another on N5D3; transcript levels (of Met, Kr-h1, BR-C and Tai) were measured on N5D6. Each point represents 4 biological replicates and results are expressed as the mean ± SEM; data are normalized against the dsMock-treated samples (reference value = 1), and the asterisk indicates statistically significant differences with respect to controls (p<0.05), according to the REST software tool [29]. (TIF) [file pone.0103614.s003.tif]
