# Supplementary material for: Role of Methoprene-Tolerant (Met) in Adult Morphogenesis and in Adult Ecdysis of Blattella germanica
Source: PLoS One. 2014 Jul 29;9(7):e103614. doi: 10.1371/journal.pone.0103614 (PMC4114754; doi:10.1371/journal.pone.0103614)
Supplement: Table S1 — Primers used to detect transcript levels by qPCR and those used to prepare the dsRNAs for RNAi experiments. (PDF) [file pone.0103614.s004.pdf]

Table S1 – Primers used to detect transcript levels by qPCR and those used to prepare the dsRNAs for RNAi experiments.

Primer set	Length (bp)	Forward primer (5'-3')	Reverse primer (5’-3’)	Encompassed Region
Met	58 bp	CTGTTGGGACATCAGCAGAA	GGCAGGTGATGGAGTGAAGT	Nucleotide 470 to 527 of HG965209
Kr-h1	77 bp	GCGAGTATTGCAGCAAATCA	GGGACGTTCTTTCGTATGGA	Nucleotide 493 to 569 of HE575250
BR-C	76 pb	CGGGTCGAAGGGAAAGACA	CTTGGCGCCGAATGCTGCGAT	Nucleotide 699 to 774 of FN651774
EcR	163 bp	GACAAACTCCTCAGAGAAGATCAAA	CTCCCAATCCTGCCAGACTA	Nucleotide 1472 to 1634 of AM039690
RXR	86 pb	ATAATTGACAAGAGGCAGAGGAA	TGAACAGCCTCCCTCTTCAT	Nucleotide 527 to 612 of AJ854490
E75A	101 bp	GTGCTATTGAGTGTGCGACATGAT	TCATGATCCCTGGAGTGGTAGAT	Nucleotide 58 to 158 of AM238653
ILP-1	96 bp	AGAAGCAGAATTCCCTTTCCG	TCATCGACAATGCCTCCGT	Nucleotide 343 to 438 of HG972850
Actin	213 pb	AGCTTCCTGATGGTCAGGTGA	TGTCGGCAATTCCAGGGTACATGGT	Nucelotide 96 to 308 of AJ862721
dsMet-a	458 pb	GCAAATTGTATCCTTCATCTGC	TGACAGACTCGCGCTTTATG	Nucelotide 1309 to 1766 of HG965209
dsMet-b	394 pb	GCAGGCGACCAAGAGTCTAC	CCACGGCAATCAGAACAGTA	Nucelotide 581 to 974 of HG965209
dsMock	307 pb	ATCCTTTCCTGGGACCCGGCA	ATGAAGGCTCGACGATCCTA	Nucelotide 370 to 676 of K01149
