# Supplementary material for: Role of Methoprene-Tolerant (Met) in Adult Morphogenesis and in Adult Ecdysis of Blattella germanica
Source: PLoS One. 2014 Jul 29;9(7):e103614. doi: 10.1371/journal.pone.0103614 (PMC4114754; doi:10.1371/journal.pone.0103614)
Supplement: Table S2 — Summary of the effects of Met depletion at phenotypic level in the experiments treating with dsMet-a or dsMet-b and at the stages N4, N5 or N6. In all cases the corresponding dsRNA was administered in two 3 µg-doses, one on day 0 and the other on day 3 of the given instar. Controls were equivalently treated with dsMock. Methodological details are described in the main text. (PDF) [file pone.0103614.s005.pdf]

Table S2 –Summary of the effects of Met depletion at phenotypic level in the experiments treating with dsMet-a or dsMet-b and at the stages N4, N5 or N6. In all cases the corresponding dsRNA was administered in two 3µg-doses, one on day 0 and the other on day 3 of the given instar. Controls were equivalently treated with dsMock. Methodological details are described in the main text.

	**Instar of treatment**	**N**	**Died before N6**	**Died during the ecdysis to N6**	**Nymphoid with adult features in N6**	**Precocious adult in N6**	**Died in ecdysis from N6 to adult**	**Adult with wrinkled wings after N6**	**Normal adult after N6**
dsMet-a	N4	54	9	8	**7**	**11**	10	4	5
dsMet-b	N4	10	1	2	**2**	**1**	1	2	1
dsMock	N4	27	0	0	**0**	**0**	0	0	27
dsMet-a	N5	35	0	0	**5**	**1**	5	12	12
dsMet-b	N5	25	0	0	**5**	**0**	1	12	7
dsMock	N5	19	0	0	**0**	**0**	0	0	19
dsMet-a	N6	28	-	-	**-**	**-**	5	10	13
dsMock	N6	14	0	0	**0**	**0**	0	0	14
